# Prevalence of type 2 diabetes, prediabetes, and gestational diabetes mellitus in women of childbearing age in Middle East and North Africa, 2000–2017: protocol for two systematic reviews and meta-analyses

**DOI:** 10.1186/s13643-018-0763-0

**Published:** 2018-07-18

**Authors:** Rami H. Al-Rifai, Faisal Aziz

**Affiliations:** Institute of Public Health, College of Medicine and Health Sciences, United Arab Emirates University, P.O. Box 17666, Al Ain, UAE

**Keywords:** Systematic review, Meta-analysis, Type 2 diabetes mellitus, Gestational diabetes mellitus, Middle East and North Africa region, MENA region

## Abstract

**Background:**

Diabetes mellitus (DM) in women of childbearing age may affect the fetus, thereby accelerating the intergenerational risk of DM. The Middle East and North Africa (MENA) region is experiencing a growing epidemic of DM. We aim to conduct two systematic reviews to summarize the burden of DM in women of childbearing age in the MENA region. In the systematic review 1, we aim to (1) systematically aggregate the evidence on type 2 DM (T2DM) and prediabetes and (2) to synthesize overall estimate on the prevalence of T2DM and overall estimate on the prevalence of prediabetes, in women of childbearing age (15–49 years). In the systematic review 2, we aim to (1) systematically aggregate the evidence on gestational DM (GDM) and (2) to synthesize overall estimate on the prevalence of GDM in pregnant women.

**Methods:**

For systematic reviews 1 and 2, we will conduct a comprehensive search of the literature published in six electronic databases (MEDLINE-PubMed, EMBASE, Web of Science, SCOPUS, Cochrane Library, and Academic Search Complete). Variant and broad search terms will be designed to identify epidemiologic studies on the prevalence of T2DM and prediabetes in women of childbearing age, and GDM in pregnant women, published between 2000 and 2017. The MENA region will be defined according to the World Bank Country and Lending Groups. Retrieved citations will be screened, and data from the eligible research reports against specific eligibility criteria will be extracted. The findings of each systematic review will be reported separately following the Preferred Reporting Items for Systematic Reviews and Meta-Analyses Guidelines (PRISMA).

**Discussion:**

Published literature on the prevalence of different types of DM among different population groups has been recently increased in the MENA region. This is the first review to fill an evidence gap on the overall burden, in the form of prevalence, of T2DM, prediabetes, and GDM in women of childbearing age in the MENA region. The findings of the two reviews will help in understanding the regional burden of these three types of DM in specific population groups to identify priority areas for interventions.

**Systematic review registration:**

The PROSPERO registration number for the systematic review 1 is “CRD42017069231” dated 12/06/2017 and for the systematic review 2 is “CRD42018100629” dated 18/06/2018.

**Electronic supplementary material:**

The online version of this article (10.1186/s13643-018-0763-0) contains supplementary material, which is available to authorized users.

## Background

Globally, non-communicable diseases (NCDs) are expected to contribute to over three quarters of all deaths in 2030 [1]. Diabetes mellitus (DM) ranks seventh of the NCDs world’s biggest killers [1]. Worldwide, the number of individuals with DM is projected to rise by 48%, from 425 million in 2017 to 629 million by 2045 [2]. Pronounced rise in the DM prevalence is projected to be in low- and middle-income countries including the African region (by 156%, from 16 million in 2017 to 41 million in 2045), the South-East Asian region (by 84%, from 82 million in 2017 to 151 million in 2045), followed by the Middle East and North African (MENA) region (by 72%, from 39 million in 2017 to 67 million in 2045) [2]. One of the global targets is to halt, by 2025, the rise in the age-standardized adult prevalence of DM at its 2010 level [3].

Type 2 DM (T2DM) is the most common type of DM, accounting for around 90% of all DM cases [4–6]. As the incidence of T2DM continues to rise and increasingly affects individuals of all ages [2], women of childbearing age, defined by the World Health Organization (WHO) as women aged between 15 and 49 years [7], are also affected by the global rise in DM epidemic. Gestational DM (GDM) affects pregnant women usually during the second and third trimesters of pregnancy though it can occur at any time during pregnancy [2]. Children of pregnant mothers with GDM are at a high risk of developing T2DM in adulthood [8], high blood pressure, or macrosomia [2]. It has been estimated that 75–90% of cases of hyperglycemia during pregnancy are related to GDM [9].

The burgeoning epidemics of T2DM and GDM have been attributed to rising levels of different modifiable risk factors: overweight and obesity driven by changes in lifestyle, a poor diet, and physical inactivity; smoking; and increase in consumption of sugar-sweetened beverages [10–13]. Worldwide, in 2016, more than 1.9 billion adults (aged ≥ 18 years) were overweight (BMI > 25 kg/m^2^); of these, over 650 million were obese (BMI > 30 kg/m^2^) [14]. An increase in the BMI is associated with an increased risk of developing T2DM or GDM [13, 15]. The overall relative risk of developing T2DM in obese and overweight individuals compared to those with a normal weight was estimated to be 7.19 and 2.99, respectively [16].

### Rationale

In the MENA region that comprises 21 countries according to the World Bank and Lending Groups [17], women show a higher prevalence of overweight and obesity than men [18]. In the MENA region in 2013, the combined age-standardized prevalence of overweight and obesity among women ≥ 20 years was 65.5% (obese 33.9%) and among men ≥ 20 years was 58.5% (obese 20.3%) [18]. This high prevalence of overweight and obesity among women may affect and exacerbate the burden of T2DM and GDM in women, eventually increasing the risk of T2DM- and GDM-associated complications and pregnancy outcomes [8, 19].

Valid and consistent estimates of T2DM, prediabetes, and GDM prevalence among specific population groups in the MENA region are needed to develop effective interventions. Estimating the burden of T2DM and prediabetes in women of childbearing age, and GDM in pregnant women at regional level in adherence to the Guidelines for Accurate and Transparent Health Estimates Reporting (GATHER) [20], requires systematic reviews and meta-analyses of studies on the regional prevalence of T2DM and prediabetes in women of childbearing age and GDM in pregnant women.

Evidence on the rising burden of T2DM and prediabetes epidemics in women of childbearing age and GDM in pregnant women in the MENA region based on individual studies will be compiled and summarized in two systematic reviews and meta-analyses studies. The findings of the two reviews, for the first time, will fill an evidence gap to inform policy makers on the epidemiologic burden of T2DM, prediabetes, and GDM in childbearing age women to design tailored interventions. Here, we present the protocol for two systematic reviews to keep a clear scope for each of the two systematic reviews*.* In fact, as the methods of systematic reviews and meta-analyses studies are similar for the most part, this protocol for two systematic reviews would avoid publishing such similar and established methods in one more additional protocol. In this protocol, we refer separately to systematic reviews 1 and 2. Each of these two reviews addresses different objectives, but they will be conducted in parallel and coherently.

### Aim

The overarching aim of the systematic review 1 is to assess the regional burden, in the form of the prevalence, of T2DM and prediabetes in women of childbearing age 15–49 years while assessing the regional burden of GDM in pregnant women will be the aim of the systematic review 2, in the MENA region during the period of January, 2000, to June, 2017. The WHO criteria to diagnose different types of DM were updated over time in 1999 [21] and then in 2006 [22]. Although we are not limiting including studies based on the DM diagnosis criteria, the lower cutoff of the year 2000 will be used as studies conducted earlier may have used outdated criteria to ascertain the diagnosis of DM. To our knowledge, this is the first systematic review protocol for estimating the regional burden of T2DM, prediabetes, and GDM in women in childbearing age and pregnant women, in the MENA region.

### Objectives

#### Systematic review 1

The objectives of systematic review 1 are as follows:

1. To systematically review quantitative studies reporting the prevalence of T2DM or prediabetes in women of childbearing age in the MENA region, from January, 2000, to June, 2017.
2. To systematically synthesize the overall estimate on the prevalence of T2DM and the overall estimate on the prevalence of prediabetes in women of childbearing age in the MENA region, as reported in studies during the period January, 2000, to June, 2017.

#### Systematic review 2

The objectives of systematic review 2 are as follows:

1. To systematically review quantitative studies reporting the prevalence of GDM in pregnant women in the MENA region, as reported in studies during the period of January, 2000 to June, 2017.
2. To systematically synthesize the overall estimate on the prevalence of GDM in pregnant women in the MENA region, as reported in studies during the period of January, 2000 to June, 2017.

### Research questions

Informed by the documented increase in the global epidemic of DM, our research questions for the systematic review 1 are (1) “what is the overall prevalence of T2DM?” and (2) “what is the overall prevalence of prediabetes?”, in women of childbearing age in the MENA region, as reported in studies published during the period of January, 2000 to June, 2017. Systematic review 2 will answer the research question “what is the overall prevalence of GDM in pregnant women?”, in the MENA region, as reported in studies published during the period of January, 2000, to June, 2017.

## Methods/design

### Description of the outcomes

The weighted mean prevalence of the following three outcomes will be assessed from studies to be included in the two systematic reviews.

#### Systematic review 1

The T2DM and prediabetes in women of childbearing age in the MENA region are the outcomes of interests.

#### Systematic review 2

In pregnant women, when the level of hyperglycemia first detected by testing at any time during the course of pregnancy meets the criteria for diagnosis of DM in the non-pregnant women, the condition is called DM in pregnancy [23]. DM in pregnancy is more likely to be detected as early as the first trimester. When hyperglycemia detected generally in the second trimester between 24 and 28 weeks of pregnancy does not meet the criteria of DM in pregnancy it is called GDM [23]. The GDM in pregnant women in the MENA region is the outcome of interest for this systematic review.

### Protocol and registration

This protocol adheres to the Preferred Reporting items for Systematic Review and Meta-Analysis Protocols (PRISMA-P) statement (Additional file 1: Table S1) [24]. The two systematic reviews will be reported according to the PRISMA statement [25]. The protocol for systematic review 1 is registered online on PROSPERO “CRD42017069231” dated 12/06/2017 and for systematic review 2 “CRD42018100629” dated 18/06/2018, the international prospective register of systematic reviews.

### Eligibility criteria

For both systematic reviews, we will include all epidemiologic studies reporting quantitative prevalence estimates according to the following inclusion and exclusion criteria:

Inclusion criteria

1. *Study design*: Observational epidemiologic studies including cross-sectional, population-based, comparative cross-sectional, and cohort studies.
2. *Population*: Women of childbearing age (15–49 years), regardless of the sample size and pregnancy status.
3. *Geographical region*: Any of the 21 countries of the MENA region, according to the definition of the World Bank Country and Lending Groups [17].
4. *Language*: Arabic or English.
5. *DM ascertainment*: Based on glucose level testing, medical records, or self-report supported with anti-DM medications or a documented diagnosis.
6. *Setting:* No limitations. Hospital-based, population-based, or clinic-based.
7. *Publication period*: January, 2000, to June, 2017.
  Exclusion criteria
8. *Study design*: Case-control, qualitative, and modeling studies; case reports and case series regardless of the number of cases; narrative reviews; conference abstracts with no full information or if authors have not responded to our inquiry on the full data; editorials; commentaries; letters to the editor; author replies; and other publications that do not include quantitative data on the prevalence of T2DM, prediabetes, or GDM.
9. *Population*: Women age less than 15 years or older than 49 years. Studies in men and studies that combined men and women together.
10. *Geographical region*: Any country not located in the MENA region, according to the definition of the World Bank Country and Lending Groups [17].
11. *Language*: Non-Arabic or non-English.
12. *DM ascertainment*: Self-report not supported with anti-DM medications or a documented diagnosis.
13. *Duplicate studies*: Studies duplicating T2DM, prediabetes, or GDM ascertainment in the same population. In the case of duplicate publications, only the study containing the most information in the context of prevalence and ascertainment methodologies will be included.
14. Studies presenting contradictory/unclear quantitative measures that could not be verified with authors.
15. Studies including patients with T2DM, prediabetes, or GDM with other metabolic disorders or other NCDs in same category.

### Information sources

For both systematic reviews, potentially eligible published articles (thereafter defined as a “research report”) will be identified through searches in six electronic academic databases (MEDLINE, EMBASE, Web of Science, SCOPUS, Cochrane library, and Academic Search Complete; see Additional file 2: Box S1). The reference lists of retrieved eligible research reports for each of the two systematic reviews will be hand-searched for further studies that might have been missed.

### Search strategy

A comprehensive and sensitive computerized search limited to the countries of the MENA region will be implemented to identify eligible published research reports for each of the two systematic reviews. We will use specific search terms based on Medical Subject Headings (MeSH) and free-text (Text) search terms. The search terms will include variant terms on the DM combined using Boolean operators. The names of individual countries of the MENA region and regional variants will also be used to identify studies that may have been indexed under regional names. To ensure the comprehensiveness of the search strategy and not to miss any potential eligible studies, expert librarians in the National Medical Library at the United Arab Emirates University were consulted on the designed search strategy and on the searching process of the six targeted databases. A detailed search strategy with filters to be applied for both systematic reviews is presented in Additional file 2: Box S1.

### Screening strategy

For both systematic reviews, the initial selection criteria will be broad to ensure that as many research reports as possible are assessed based on their relevance to each of the two systematic reviews. Any research report that are not relevant to any of the two systematic reviews will be excluded in the early stages of the title and abstract screening. The full-text research reports of seemingly relevant titles/abstracts will be accessed online. Research reports that are not freely available online will be obtained through the University Interlibrary Loan System. The full texts of research reports will be read for further screening based on the eligibility criteria. Two authors will independently screen the titles, abstracts, and full texts of potentially relevant research reports. Any conflicts between the two authors will be resolved by consensus or by consulting an expert.

#### Removal of duplicates

For both systematic reviews, the Endnote X8 reference manager database [26] will be used to manage the retrieval of articles and to screen for and exclude the duplicate reports. The following rules will be used to remove duplicate hits from the database:

1. Compare the title or various combinations of the author(s), year, secondary title, volume, issue, and page numbers with the “de-duplication” function.
2. Visually compare the full records of suspected duplicates.
3. Save duplicates in a separate database.

#### First-level screening—title/abstract screening

All titles/abstracts will be screened for relevance. Screened titles/abstracts will be classified into three categories: not relevant, relevant, or potentially relevant.

#### Second-level screening—full-text screening

The full texts of potentially relevant and completely relevant research reports identified during first-level screening will be retrieved and thoroughly evaluated to assess their eligibility. Irrelevant research reports against our eligibility criteria will be excluded.

### Data extraction

For research reports found eligible to be included in systematic review 1 and systematic review 2, relevant data will be extracted into a predefined data extraction form, which will first be piloted using five research reports. The data will be extracted independently by two review investigators. Discrepancies between data extractors will be discussed and resolved to reach a consensus. If a consensus cannot be achieved, an expert will be consulted.

The following parameters will be extracted from relevant studies: author names, date of publication, country where the study was conducted, number of participants, year of publication, study design, setting, target population, study period, methodology of outcome ascertainment, number of subjects enrolled, number of the tested women, age of the tested women, pregnant or child-bearing age, number of the women diagnosed with T2DM, prediabetes, or GDM, and prevalence of T2DM, prediabetes, or GDM. A complete list of the coded parameters to be extracted for both systematic reviews is shown in Additional file 2: Table S2.

### Data synthesis

For both systematic reviews, research reports providing stratified estimates of the prevalence, the prevalence of the total sample will be replaced with pooling the stratified measures. A pre-defined sequential order will be followed when considering stratified prevalence measures. Prevalence estimates stratified according to BMI will be prioritized, followed by age, and year. This prioritization scheme will prioritize strata with more information on the tested subjects. Otherwise, the overall T2DM, prediabetes, or GDM prevalence measure will be included. In research reports stratifying prevalence of the outcome of interest at different levels (i.e., age and BMI), one stratification level per included research report will be considered and included to avoid double-counting. If T2DM, prediabetes, or GDM was diagnosed using various ascertainment criteria, we will extract relevant information of the most sensitive and reliable ascertainment assay (i.e., prioritizing FBG over self-reported DM status).

### Risk of bias assessment

We will evaluate the precision and the quality of each prevalence estimate measure identified in each of the two systematic reviews. We will assess each study’s risk of bias (ROB) using the NIH tool for observational studies that has 14 criteria [27]. Also, we will use three additional quality criteria, namely, sampling methodology, ascertainment methodology, and response rate. These three additional criteria will assess the robustness of the implemented sampling methodology and the ascertainment methodology of the measured outcome(s). For each assessed criteria, each study has the potential to be categorized as “potentially of low ROB” if the answer was “yes” for that specific criteria, “potentially of high ROB” if the answer was “no” for that specific criteria, or “unclear” if the answer was neither “can’t determine or not reported” for that specific criteria. A low ROB will be considered if the study/research report evaluated as a probability based, ascertained by biological assays, or with a response rate of ≥ 80%.

To assess the precision of the reported prevalence estimates, a minimum sample size will be calculated using an established mathematical formula [28]. Each prevalence measure will be considered as having a high precision if the tested sample size was equal or higher than the calculated minimum sample size [28]. Items included to measure the ROB are listed in Additional file 2: Table S2. The ROB and precision will be reported for each research report not for each prevalence measure, unless different prevalence measures within the same research report were based on different methodologies such as a different sampling strategy.

### Summary measures and synthesis of results: meta-analysis

For each systematic review, we will use the *metaprop* command to perform a meta-analysis of the prevalence estimates [29]. This command incorporates the Freeman-Tukey double arcsine transformation to stabilize the variances of prevalence measures [30, 31]. The prevalence measures will be weighted using the inverse variance method [31]. For each pooled estimate and its 95% confidence interval (CI), a forest plot will be created to show the prevalence and corresponding 95% CI for each study.

Several guidelines are available and implemented to ascertain different types of DM including the guidelines issued by the WHO in 1999 [21], WHO in 2006 [22], American Diabetes Association [32, 33], Carpenter and Coustan criteria [34], International Association of Diabetes and Pregnancy Study Groups [35], and National Diabetes Data Group Classification [36]. For both systematic reviews, the estimation of the pooled prevalence will be performed according to the ascertainment methodology and overall regardless of the ascertainment methodology. All analyses will be performed using the STATA SE15.0 statistical software [37].

For each systematic review, the heterogeneity in effect size will be evaluated across studies. Specifically, we will conduct Cochran’s *Q* test and extract several heterogeneity measures, including the estimate of the between-study variance of the true effect sizes using the *τ*-squared statistic (*τ*^2^), the magnitude and its 95% CI of the between-study variation that is due to heterogeneity rather than chance using the *I*-squared statistic (*I*^*2*^), and the 95% prediction interval which estimates the distribution of the true effect size among the included studies [38, 39]. The *Q*-statistic tests for heterogeneity are based on the null hypothesis that all studies share a common effect size. Hypothesis testing will be performed based on a *p* value < 0.10, implying that the studies do not share a common effect size [39].

### Assessment of meta-bias

For each systematic review, we will assess the presence of meta-bias in terms of publication bias. To assess the publication bias, we will examine funnel plots supplemented with formal statistical testing using Egger’s test [40]. The funnel plots of log (odds proportion) will be plotted against the sample size [41]. The asymmetry of the funnel plots will be examined by performing Egger’s test [40] that is based on SE. To test the robustness of our findings, we will apply Duval and Tweedie’s trim and fill methods [42].

### Additional analyses

In each systematic review, if there is evidence for differences in prevalence estimates measures, the source of heterogeneity will be explored through subgroups analyses, according to DM-ascertainment criteria, using study-level characteristics such as sub-region, study period, year of publication, and precision. The MENA region will be divided into four sub-regions, including the North Africa region (Morocco, Algeria, Tunisia, Libya, Egypt, Malta, and Djibouti); Levant region (Jordan, Syria, Palestine, Israel, and Lebanon), Gulf Cooperation Council countries (United Arab Emirates, Qatar, Saudi Arabia, Kuwait, Yemen, Oman, and Bahrain), as well as Iran and Iraq. If feasible, sensitivity analyses will be conducted for studies with high precision or a low ROB. This may be complemented, where relevant, by meta-regression to further explain the heterogeneity. To construct a multivariable meta-regression, we will first evaluate the crude contribution of each factor to the log odds of the outcome prevalence at *p* < 0.10. Then, we will sequentially add variables to the meta-regression starting with the variables showing the strongest correlation with the estimated prevalence separately, based on the univariate meta-regression analyses and relevance. The final regression model will include all variables that are significantly and independently associated with each of the measured three outcomes [38].

## Discussion

Globally, the growing epidemic of diabetes and its increasing penetration in women of childbearing age have generated public health concerns on adverse pregnancy outcomes and accelerating the intergenerational risk of DM. The two systematic reviews to be conducted, to the best of our knowledge, will be the first to synthesis available data on T2DM and prediabetes in women of childbearing age and GDM in pregnant women, in the MENA region. Findings of the two reviews will fill an evidence gap in understanding the regional burden of T2DM, prediabetes, and GDM among specific population groups. The two systematic reviews will provide basis regional estimates for the future epidemiologic studies. Additionally, the two reviews will inform policy makers prioritize areas for interventions to improve maternal and child health outcomes in the MENA region.

## Additional files

Additional file 1: **Table S1.** PRISMA-P 2015 checklist. Prevalence of type 2 diabetes, prediabetes, and gestational diabetes mellitus in women of childbearing age in Middle East and North Africa, 2000–2017: protocol for two systematic reviews and meta-analyses. (DOCX 33 kb)

Additional file 2: **Box S1.** Databases and search terms*.* **Table S2.** Data extraction parameters. (DOCX 44 kb)

## Abbreviations

BMI: Body mass index
CI: Confidence interval
DM: Diabetes mellitus
FBG: Fasting blood glucose
GATHER: Guidelines for Accurate and Transparent Health Estimates Reporting
GDM: Gestational diabetes mellitus
*I*^2^: *I*-squared statistic
IGR: Impaired glucose regulation
IGT: Impaired glucose tolerance
MENA: Middle East and North Africa
NCDs: Non-communicable diseases
OGTT: Oral glucose tolerance test
Prediabetes: Prediabetes mellitus
PRISMA: Preferred reporting items for systematic reviews and meta-analyses guidelines
PRISMA-P: Preferred Reporting Items for Systematic Review and Meta-Analysis Protocols
ROB: Risk of bias
T2DM: Type 2 diabetes mellitus
UN: United Nations
WHO: World Health Organization
*τ*^2^: *τ*-squared statistic
