# Supplementary material for: Prevalence of type 2 diabetes, prediabetes, and gestational diabetes mellitus in women of childbearing age in Middle East and North Africa, 2000–2017: protocol for two systematic reviews and meta-analyses
Source: Syst Rev. 2018 Jul 18;7:96. doi: 10.1186/s13643-018-0763-0 (PMC6052625; doi:10.1186/s13643-018-0763-0)
Supplement: Supplementary file 1 — Table S1. PRISMA-P 2015 checklist. Prevalence of type 2 diabetes, prediabetes, and gestational diabetes mellitus in women of childbearing age in Middle East and North Africa, 2000–2017: protocol for two systematic reviews and meta-analyses. (DOCX 33 kb) [file 13643_2018_763_MOESM1_ESM.docx]

**Table S1. PRISMA-P 2015 checklist.** Prevalence of type 2 diabetes, prediabetes, and gestational diabetes mellitus in women of childbearing age in Middle East and North Africa, 2000–2017: protocol for two systematic reviews and meta-analyses

| Section and topic | Item No | Checklist item | Reported on page # of main text |
| --- | --- | --- | --- |
| ADMINISTRATIVE INFORMATION | | |  |
| Title: |  |  |  |
| Identification | 1a | Identify the report as a protocol of a systematic review | Page 1 |
| Update | 1b | If the protocol is for an update of a previous systematic review, identify as such | Not applicable |
| Registration | 2 | If registered, provide the name of the registry (such as PROSPERO) and registration number  *The study has been registered under PROSPERO. The PROSPERO registration number is CRD42017068910* | Page 4 |
| Authors: |  |  |  |
| Contact | 3a | Provide name, institutional affiliation, e-mail address of all protocol authors; provide physical mailing address of corresponding author | Page 1 |
| Contributions | 3b | Describe contributions of protocol authors and identify the guarantor of the review | Page 19 |
| Amendments | 4 | If the protocol represents an amendment of a previously completed or published protocol, identify as such and list changes; otherwise, state plan for documenting important protocol amendments  *The protocol is not an amendment.* | Not applicable |
| Support: |  |  |  |
| Sources | 5a | Indicate sources of financial or other support for the review | Page 19 |
| Sponsor | 5b | Provide name for the review funder and/or sponsor | Page 19 |
| Role of sponsor or funder | 5c | Describe roles of funder(s), sponsor(s), and/or institution(s), if any, in developing the protocol | Not applicable |
| INTRODUCTION | | |  |
| Rationale | 6 | Describe the rationale for the review in the context of what is already known | Pages 5-6 |
| Objectives | 7 | Provide an explicit statement of the question(s) the review will address with reference to participants, interventions, comparators, and outcomes (PICO) | Page 7-8 |
| METHODS | | |  |
| Eligibility criteria | 8 | Specify the study characteristics (such as PICO, study design, setting, time frame) and report characteristics (such as years considered, language, publication status) to be used as criteria for eligibility for the review | Page 9-10 |
| Information sources | 9 | Describe all intended information sources (such as electronic databases, contact with study authors, trial registers or other grey literature sources) with planned dates of coverage | Page 11 |
| Search strategy | 10 | Present draft of search strategy to be used for at least one electronic database, including planned limits, such that it could be repeated | Box S1 |
| Study records: |  |  |  |
| Data management | 11a | Describe the mechanism(s) that will be used to manage records and data throughout the review | Page 12-13 |
| Selection process | 11b | State the process that will be used for selecting studies (such as two independent reviewers) through each phase of the review (that is, screening, eligibility and inclusion in meta-analysis) | Page 12-13 |
| Data collection process | 11c | Describe planned method of extracting data from reports (such as piloting forms, done independently, in duplicate), any processes for obtaining and confirming data from investigators | Page 14 |
| Data items | 12 | List and define all variables for which data will be sought (such as PICO items, funding sources), any pre-planned data assumptions and simplifications | Table S2 |
| Outcomes and prioritization | 13 | List and define all outcomes for which data will be sought, including prioritization of main and additional outcomes, with rationale | Page 8 |
| Risk of bias in individual studies | 14 | Describe anticipated methods for assessing risk of bias of individual studies, including whether this will be done at the outcome or study level, or both; state how this information will be used in data synthesis | Page 15 |
| Data synthesis | 15a | Describe criteria under which study data will be quantitatively synthesised | Page 13 |
|  | 15b | If data are appropriate for quantitative synthesis, describe planned summary measures, methods of handling data and methods of combining data from studies, including any planned exploration of consistency (such as I^2^, Kendall’s τ) | Page 13-15 |
|  | 15c | Describe any proposed additional analyses (such as sensitivity or subgroup analyses, meta-regression) | Page 16 |
|  | 15d | If quantitative synthesis is not appropriate, describe the type of summary planned | Page 16 |
| Meta-bias(es) | 16 | Specify any planned assessment of meta-bias(es) (such as publication bias across studies, selective reporting within studies) | Page 16 |
| Confidence in cumulative evidence | 17 | Describe how the strength of the body of evidence will be assessed (such as GRADE) | Page 8 |
