# Supplementary material for: Prevalence of type 2 diabetes, prediabetes, and gestational diabetes mellitus in women of childbearing age in Middle East and North Africa, 2000–2017: protocol for two systematic reviews and meta-analyses
Source: Syst Rev. 2018 Jul 18;7:96. doi: 10.1186/s13643-018-0763-0 (PMC6052625; doi:10.1186/s13643-018-0763-0)
Supplement: Supplementary file 2 — Box S1. Databases and search terms. Table S2. Data extraction parameters. (DOCX 44 kb) [file 13643_2018_763_MOESM2_ESM.docx]

**Supplementary Information**

**Prevalence of type 2 diabetes, prediabetes, and gestational diabetes mellitus in women of childbearing age in Middle East and North Africa, 2000–2017: protocol for two systematic reviews and meta-analyses**

Rami H. Al-Rifai^1,*^, Faisal Aziz^1^

*^1^ Institute of Public Health, College of Medicine and Health Sciences, United Arab Emirates University, Al–Ain, United Arab Emirates, P.O. Box 15551, Al Ain. Email: rrifai@uaeu.ac.ae*

**^*^Corresponding author:**

Correspondence to: Rami H. Al-Rifai, Institute of Public Health, College of Medicine and Health Sciences, United Arab Emirates University, Al-Ain, United Arab Emirates, P.O. Box 15551, Al Ain, UAE. Telephone: +(971) 3713-7450. Fax: +(971) 3767-2022. E-mail: rrifai@uaeu.ac.ae

**Box S1: Databases and search terms**

1. **MEDLINE–PubMed**

((("Diabetes Mellitus”[Mesh] OR "Diabetes Mellitus, Type 2"[Mesh] OR “diabetes type 2”[Mesh] OR “T2DM”[Mesh] OR “diabetes Type II”[Mesh] OR diabetes”[Mesh] OR "glucose intolerance"[Mesh] OR "insulin resistance"[Mesh] OR "Hyperglycemia"[Mesh] OR "Hypoglycemia"[Mesh] OR OR "Diabetes Mellitus”[Text] OR "Diabetes Mellitus, Type 2"[Text] OR “diabetes type 2”[Text] OR “T2DM”[Text] OR “diabetes Type II”[Text] OR diabetes”[Text] OR "glucose intolerance"[Text] OR "insulin resistance"[Text] OR "Hyperglycemia"[Text] OR "Hypoglycemia"[Text]) AND ("Adolescent"[Mesh] OR "Young Adult"[Mesh] OR "Adult"[Mesh] OR "Middle Aged"[Mesh] OR “teenage”[Mesh] OR “adolescent”[Mesh] OR “young adult”[Mesh] OR “adult”[Mesh] OR “Middle Age”[Mesh] OR "Adolescent"[Text] OR "Young Adult"[Text] OR "Adult"[Text] OR "Middle Aged"[Text] OR “teenage”[Text] OR “adolescent”[Text] OR “young adult”[Text] OR “adult”[Text] OR “Middle Age”[Text]) AND ("Middle East"[Mesh] OR "Islam"[Mesh] OR "Arabs"[Mesh] OR "Arab World"[Mesh] OR "Africa, Northern"[Mesh] OR "Djibouti"[Mesh] OR "Pakistan"[Mesh] OR "Middle East"[Text] OR "Middle-East"[Text] OR "North Africa"[Text] OR "North-Africa"[Text] OR "EMRO"[Text] OR "Eastern Mediterranean"[Text] OR “Arab”[Text] OR “Arabs”[Text] OR “Arab World”[Text] OR "Islam"[Text] OR "Afghanistan"[Text] OR "Algeria"[Text] OR "Bahrain"[Text] OR "Djibouti"[Text] OR "Egypt"[Text] OR "Jordan"[Text] OR "Kuwait"[Text] OR "Lebanon"[Text] OR "Libya"[Text] OR "Iran"[Text] OR "Iraq"[Text] OR "Morocco"[Text] OR "Oman"[Text] OR "Pakistan"[Text] OR "Qatar"[Text] OR "Saudi Arabia"[Text] OR "Syria"[Text] OR "Tunisia"[Text] OR "United Arab Emirates"[Text] OR "Dubai"[Text] OR "Abu Dhabi"[Text] OR "Abu-Dhabi"[Text] OR “Sharjah”[Text] OR "West Bank"[Text] OR "Ghaza"[Text] OR "Palestine"[Text] OR "Yemen"[Text])))

**Search filters:** Human studies; Year: 2000-2017

1. **Embase**

(((‘Diabetes Mellitus, Type 2’/exp OR ‘Diabetes Mellitus, Type II'/exp OR ‘diabetes type 2’/exp OR ‘T2DM’/exp OR ‘diabetes Type II’/exp OR ‘diabetes’/exp OR ‘hyperglycemia’/exp OR ‘insulin resistance’/exp OR ‘hypoglycemia’/exp OR ‘oral glucose tolerance’/exp)) AND ((‘Adolescent’/exp OR ‘Young Adult’/exp OR ‘Adult’/exp OR ‘Middle Aged’/exp OR teenage* OR adolescen*)) AND ((‘Middle East’/exp OR ‘North Africa’/exp OR ‘Arab’/exp OR ‘Afghanistan’/exp OR ‘Djibouti’/exp OR ‘Pakistan’/exp OR ‘EMRO’/exp OR ‘Eastern Mediterranean’/exp OR ‘Arabs’/exp OR ‘Arab World’/exp OR ‘Islam’/exp OR ‘Algeria’/exp OR ‘Bahrain’/exp OR ‘Egypt’/exp OR ‘Jordan’/exp OR ‘Kuwait’/exp OR ‘Lebanon’/exp OR ‘Libya’/exp OR ‘Iran’/exp OR ‘Iraq’/exp OR ‘Morocco’/exp OR ‘Oman’/exp OR ‘Qatar’/exp OR ‘Saudi Arabia’/exp OR ‘Syria’/exp OR ‘Tunisia’/exp OR ‘United Arab Emirates’/exp OR ‘Dubai’/exp OR ‘Abu Dhabi’/exp OR ‘Sharjah’/exp OR ‘West Bank’/exp OR ‘Ghaza’/exp OR ‘Palestine’/exp OR ‘Yemen’/exp)))

**Search filters:** human studies; year: 2000-2017; Publication type (Article; article in press; Conference Abstract; Conference Paper; Conference Review; Language: English, Arabic; Gender: Female.

1. **WEB OF SCIENCE**

TS= (Diabete* OR T2DM OR Hyperglycemia OR "insulin resistance" OR Hypoglycemia OR "glucose intolerance") AND TS= (Adolescen* OR "Young Adult" OR Adult OR "Middle Aged" OR teenage*) AND TS= ("Middle East" OR "North Africa" OR Arab OR Afghanistan OR Djibouti OR Pakistan OR EMRO OR "Eastern Mediterranean" OR Arabs OR "Arab World" OR Islam OR Algeria OR Bahrain OR Egypt OR Jordan OR Kuwait OR Lebanon OR Libya OR Iran OR Iraq OR Morocco OR Oman OR Qatar OR "Saudi Arabia" OR Syria OR Tunisia OR "United Arab Emirates" OR Dubai OR "Abu Dhabi" OR Sharjah OR "West Bank" OR Ghaza OR Palestine OR Yemen)

**Search filters:** Language: English OR Arabic; document types: Article OR Abstract of Published Item OR Meeting Abstract OR Meeting Summary OR Proceedings Paper; Timespan: 2000-2017

1. **SCOPUS**

((TITLE-ABS-KEY(diabete! OR t2dm OR hyperglycemia OR "insulin resistance" OR hypoglycemia OR "glucose intolerance") AND TITLE-ABS-KEY(adolescen! OR “Young Adult” OR adult OR “Middle Aged” OR teenage!) AND TITLE-ABS-KEY("Middle East" OR "North Africa" OR Arab OR Afghanistan OR Djibouti OR Pakistan OR Bahrain OR Egypt OR Jordan OR Kuwait OR Lebanon OR Libya OR Iran OR Iraq OR Morocco OR Oman OR Pakistan OR Qatar OR Saudi Arabia OR Syria OR Tunisia OR United Arab Emirates OR Dubai OR Abu Dhabi OR Sharjah OR West Bank OR Ghaza OR Palestine OR Yemen))

**Search filters:** Search Field: Title-Abs-Key; Pubyear: 2000 to 2017; limit-to doctype: Articles or Conference Paper; limit-to language: English or Arabic.

1. **COCHRANE LIBRARY**

(Diabete* or T2DM or *glycemia or insulin resistan* or glucose intoleran*) AND (Adolescen* or "Young Adult" or Adult or "Middle Aged" or teenage*) AND ("Middle East" or "North Africa" or Arab or Afghanistan or Djibouti or Pakistan or EMRO or "Eastern Mediterranean" or Arabs or "Arab World" or Islam or Algeria or Bahrain or Egypt or Jordan or Kuwait or Lebanon or Libya or Iran or Iraq or Morocco or Oman or Qatar or "Saudi Arabia" or Syria or Tunisia or "United Arab Emirates" or Dubai or "Abu Dhabi" or Sharjah or "West Bank" or Ghaza or Palestine or Yemen)

**Search filters:** Search Field: All Text; Publication Year: 2000 to 2017.

1. **ACADEMIC SEARCH COMPLETE**

((Diabete* OR T2DM OR Hyperglycemia OR "insulin resistance" OR Hypoglycemia OR "glucose intolerance") AND (Adolescen* OR "Young Adult" OR Adult OR "Middle Aged" OR teenage*) AND ("Middle East" OR "North Africa" OR Arab OR Afghanistan OR Djibouti OR Pakistan OR EMRO OR "Eastern Mediterranean" OR Arabs OR "Arab World" OR Islam OR Algeria OR Bahrain OR Egypt OR Jordan OR Kuwait OR Lebanon OR Libya OR Iran OR Iraq OR Morocco OR Oman OR Qatar OR "Saudi Arabia" OR Syria OR Tunisia OR "United Arab Emirates" OR Dubai OR "Abu Dhabi" OR Sharjah OR "West Bank" OR Ghaza OR Palestine OR Yemen))

**Search filters**

Limiters: References Available; Scholarly (Peer Reviewed) Journals.

Published Date: 2000-2017.

Document Type: Abstract, Article, Bibliography, Proceeding.

Language: Arabic, English

Search modes: Boolean/Phrase

Select a Field: All Text

**Table S2**. Data extraction parameters.

| **Variable Name** | **Variable Type** | **Variable Label** | **Values** |
| --- | --- | --- | --- |
| ReportNo | Numeric | Sequential report number (1,2,….) | Numeric |
| StudyNo | Numeric | Sequential study number (1,2,….) | Numeric |
| ID | Numeric | Study ID | Numeric |
| Author | String | First author’s name, initials, et al | String |
| Pub_year | Numeric | Publication year | Numeric |
| Article_type | String | Type of the publication | 1. Original article 2. Conference abstract 3. Short communication 4. Letter |
| Journal | String | Journal name | String |
| Country | String | Country where the study was executed | String |
| Region | Numeric | Exact region within MENA | 1. North Africa (Morocco, Algeria, Tunisia, Libya, Egypt, Malta, Djibouti) 2. Levant (Jordan, Syria, Palestine/Israel, Lebanon) 3. Arabian Gulf (UAE, Qatar, Saudi Arabia, Kuwait, Yemen, Oman, Bahrain) 4. Iraq/Iran |
| City | String | City where the study was executed | String |
| Study_des | Numeric | Study design | 1. Cross-sectional 2. Prospective cohort 3. Retrospective cohort 4. Unclear |
| Yrstart | Date (MM/DD/YYYY) | Year data collection started | Numeric |
| Yrend | Date (MM/DD/YYYY) | Year data collection ended | Numeric |
| Duration | Numeric | Data collection duration in months | Numeric |
| Location | Numeric | Location from where the study population was chosen | 1. General population/National health database/national surveys 2. Hospitals 3. Outpatient clinics 4. Elderly health service center 5. Organ transplantation centers 6. Unclear |
| Population | Numeric | Studied population | 1. Pregnant women 2. Dialysis patients 3. Arthritis patients 4. Kidney transplant 5. Liver transplant 6. Other organ transplant 7. Health care workers 8. General population 9. Cancer patients 10. HIV patients 11. COPD patient 12. Schizophrenic patients 13. Unclear |
| Sampling_strategy | Numeric | Subjects sampling strategy | 1. Convenience 2. Systematic 3. Random sampling 4. Multistage probability sampling 5. Whole populations (e.g. all dialysis patients) 6. Unclear |
| Marital | Numeric | Marital status of the women of childbearing age | 1. Married 2. Single 3. Unclear |
| Age_measure | Numeric | Reported age measure | 1. Mean 2. Median 3. Range |
| Age | String | Mean, median, or age range of women | Numeric |
| DM_awareness | Numeric | Women were aware on their DM status (e.g. previously diagnosed with DM) | 1. Yes (they were aware) 2. No (they were not aware) 3. Unclear |
| Pregnancy | Numeric | Pregnancy status at time to DM ascertainment | 1. Pregnant 2. Not pregnant (childbearing age women) 3. Unclear |
| Pregnancytrim | Numeric | Pregnancy trimester | 1. First 2. Second 3. Third 4. Unclear |
| DM_Dx | Numeric | Specific method of DM ascertainment assay | 1. Self-reported confirmed by anti-DM therapies or documented diagnosis 2. Fasting blood glucose (FBG) 3. HbA1c (Glycated hemoglobin) 4. **Random blood sugar** 5. **Oral glucose tolerance test (OGTT)** 6. **Medical records** 7. **Unclear** |
| DM_Dx_Criterai | Numeric | Exact DM diagnostic criteria or guidlines | 1. WHO 1999 2. WHO 2006 3. Carpenter and Coustan 4. American diabetes association 5. **NICE Guidelines** 6. **I**nternational Association of Diabetes and Pregnancy Study Groups (IADPSG) 7. Unclear (e.g. self-reported or medical records) |
| DM_Dx description | String | Description of DM diagnosis process with the cut-off point as reported in the study | String |
| DM_type | Numeric | Type of the diagnosed DM | 1. Type 1 DM 2. Type 2 DM (explicitly stated) 3. GDM (gestational DM) 4. Intermediate hyperglycemia/prediabetes 5. Unclear (presumably type 2 DM) |
| Sample_type | Numeric | Sample type included in the analysis | 1. Whole sample 2. Stratified |
| Sample_strata | String | Sample stratified by what (e.g. Comorbidity, age) | String (Prioritize extracting DM prevalence strata by BMI, age, and year) |
| Measure_type | Numeric | Reported measure | 1. Prevalence 2. Incidence |
| Sample_size | Numeric | Tested sample size | Numeric |
| DM_No_Prev | Numeric | Number of DM cases | Numeric |
| DM_Prev | Numeric | Prevalence of DM | Numeric |
| DM_No_Inc | Numeric | Number of NEW (incident) DM cases in cohort studies | Numeric |
| DM_Incidence | Numeric | Incidence of DM per 100,000 person-year | Numeric |
| Recruited | Numeric | Sample size at recruitment | Numeric |
| Follow_Up | String | Duration of follow-up | String |
| Lost | String | Number of individuals who were lost to follow-up in cohort studies | String |
| ROB1 | Numeric | DM ascertainment assay | 1. High ROB (Medical records) 2. Low ROB (biological assay) 3. Unclear ROB (not specified) |
| ROB2 | Numeric | Sampling methodology | 1. High ROB (Non-probability-based) 2. Low ROB (Probability-based) 3. Unclear ROB |
| ROB3 | Numeric | Response rate (tested proportion out of recruited sample) | 1. High ROB (<80%) 2. High ROB (≥80%) 3. Unclear ROB |
| ROB4 | Numeric | Precision | 1. High precision 2. Low precision 3. Unclear precision |
